# Prevalence and correlates of chronic kidney disease (CKD) among ART-naive HIV patients in the Niger-Delta region of Nigeria

**DOI:** 10.1097/MD.0000000000010380

**Published:** 2018-04-20

**Authors:** Udeme E. Ekrikpo, Andre P. Kengne, Effiong E. Akpan, Emmanuel E. Effa, Aminu K. Bello, John U. Ekott, Cindy George, Babatunde L. Salako, Ikechi G. Okpechi

**Affiliations:** a Division of Nephrology and Hypertension, Department of Medicine, University of Cape Town, Cape Town, South Africa; b Department of Medicine, University of Uyo, Uyo, Nigeria; c Non-Communicable Diseases Research Unit, South African Medical Research Council, Cape Town, Cape Town, South Africa; d Department of Medicine, University of Calabar, Calabar, Nigeria; e Division of Nephrology and Immunology, Department of Medicine, University of Alberta, Edmonton, Canada; f Department of Medicine, College of Medicine, University of Ibadan, Ibadan, Nigeria; g Kidney and Hypertension Research Unit, University of Cape Town, Cape Town, South Africa.

**Keywords:** CKD, HIV, Nigeria, prevalence

## Abstract

Widespread use of antiretroviral therapy (ART) in human immunodeficiency virus (HIV) patients has led to improved longevity with the attendant increase in noncommunicable disease prevalence including chronic kidney disease (CKD). This study documents the prevalence of CKD in a large HIV population in Southern Nigeria.

This is a single center, 15-year analysis in ART-naïve patients. CKD was defined as the occurrence of estimated glomerular filtration rate (eGFR) <60 mL/min/1.73 m^2^ on 2 consecutive occasions 3 to 12 months apart using the chronic kidney disease epidemiology collaboration (CKD-EPI) equation. The Cochran-Armitage and Cuzick tests were employed to assess for trend across the years for CKD prevalence and CD4 count, respectively. Multivariable logistic regression models were used to identify independent associations with CKD.

In all, 1317 patients (62.2% females) with mean age of 34.5 years and median CD4 count of 194 cells/μL were included. CKD prevalence was 13.4% (95%CI 11.6%–15.4%) using the CKD-EPI equation (without the race factor). Multivariable analysis identified increasing age and CD4 count <200 cells/μL as being independently associated with CKD occurrence.

This study reports a high prevalence of CKD in ART-naïve HIV-infected patients. Measures to improve diagnosis of kidney disease and ensure early initiation of treatment should be integrated in HIV treatment programmes in this setting.

## Abstract 2

Supplemental Digital Content is available in the text

## 1 Introduction

As at the end of 2015, about 36.7 million people were living with the human immunodeficiency virus (HIV) globally with 23.5 million living in Sub-Saharan Africa (SSA).^[1]^ Of those affected, 3.2 million individuals live in Nigeria with the highest prevalence reported from the Niger-Delta region.^[2]^ Nigeria is estimated to have the 2nd highest HIV population in the world, with South Africa ranking 1st with 7 million affected individuals.^[3]^ The increased availability of antiretroviral therapies (ARTs) in many SSA countries has led to increased life expectancy with an attendant increase of noncommunicable diseases among HIV populations.^[1,4]^

Kidney disease is a common complication of HIV infection^[5,6]^ and HIV is a common cause of chronic kidney disease (CKD) in SSA.^[7,8]^ Despite the rationing of dialysis in South Africa where being HIV positive adversely impacts on patients’ chances of acceptance to dialysis, the South African Renal registry has recently reported an increase from 8.3% in 2012 to 9.3% in 2014 of HIV positive end-stage renal disease patients receiving dialysis.^[9]^ One study from the US showed that the annual number of patients with incident end-stage renal disease secondary to HIV-associated nephropathy (HIVAN) increased steadily from 1989 to 1995 and then remained stable until 2006.^[10]^ Also, some studies in Nigeria have documented high prevalence of kidney disease among HIV patients ranging from 22.9% to 51.8% depending on the geographic location and definition of kidney disease utilized.^[11–14]^ Such data continue to highlight the impact of HIV on kidney disease. However, most of the studies have been underpowered and may therefore not report accurate estimates of CKD prevalence. There is therefore a need to assess kidney disease prevalence in SSA using a large HIV study population to provide a more accurate estimate of the disease burden. This will assist the planning for health services delivery in the region.

## 2 Methods

**Figure 1:**
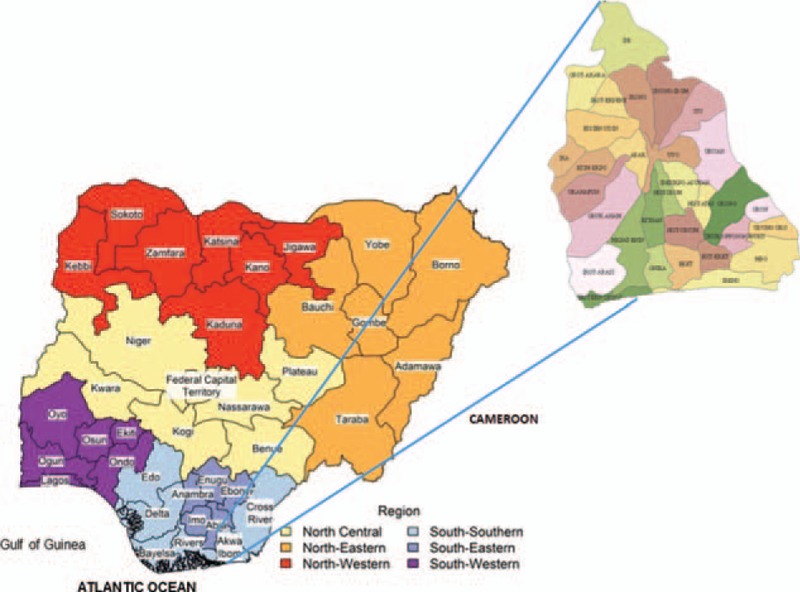
Map of Nigeria showing Akwa Ibom State.

This study is a 15-year (2002–2016) assessment of renal function in ART-naïve HIV-infected patients at the University of Uyo Teaching Hospital (UUTH), Uyo, Nigeria (Human Research Ethics number: UUTH/AD/S/vol. XIX/15. August 9, 2016). The UUTH is the only tertiary health facility serving a population of over 4 million people in the extreme Southern (Niger-Delta) region of Nigeria (Fig. 1). The UUTH HIV clinic, funded by the United States Agency for International Development, is involved in voluntary counseling and testing for HIV, provision of ART, identification and treatment of opportunistic infections, and follow-up care for HIV positive patients. Clinical and demographic features such as age, gender, weight, height, body mass index (BMI), blood pressure, hypertension and diabetes mellitus (DM) status, and date of commencement of ART were extracted from the records. Records for CD4 count, viral load, electrolytes, urea, creatinine (measured using an isotope dilution mass spectrophotometry-traceable Jaffe kinetic reaction), hepatitis B surface antigen, and antibody to hepatitis C virus were also extracted. Serum creatinine is routinely done at first contact with the patient but programmatic deficiencies do not often allow repeat serum creatinine except in those who can afford out-of-pocket payment for the test. Information on proteinuria (dipstick assessment) was also recorded where available, because this was not routinely done. Hypertension was defined as 2 or more recordings of blood pressure with systolic blood pressure (SBP) at least 140 mm Hg and/or diastolic blood pressure (DBP) of at least 90 mm Hg or patients on antihypertensive medication.^[15]^ Mean arterial blood pressure was calculated as [DBP + (SBP − DBP)/3]. DM was defined as fasting plasma glucose of at least 7.0 mmol/L and/or random/2 hour postmeal plasma glucose of at least 11.1 mmol/L^[16]^ or in patients taking antidiabetic agents. Obesity was defined as BMI of at least 30 kg/m^2^ in a patient without peripheral edema^[17]^; overweight as BMI of 25 to 29.9 kg/m^2^ while BMI of 18.0 to 24.9 and less than 18.0 kg/m^2^ were defined as normal and underweight, respectively. Dyslipidemia was defined using the National Cholesterol Education program adult treatment panel III criteria^[18]^ – total cholesterol greater than 200 mg/dL or low density lipoprotein cholesterol greater than 150 mg/dL or high density lipoprotein cholesterol less than 40 mg/dL or triglycerides greater than 150 mg/dL.

For the purposes of this study, data were extracted from patient's physical case records and transferred into STATA 14 (StataCorp, TX) for analysis. The Student *t* test (or its nonparametric equivalent, the Mann–Whitney *U* test, where necessary) was used to compare continuous variables while the Chi-square test was used to compare categorical variables. CKD was defined as 2 consecutive values of estimated glomerular filtration rate (eGFR) <60 mL/min/1.73 m^2^ recorded within 3 to 12 months apart. We estimated GFR using the chronic kidney disease epidemiology collaboration (CKD-EPI) equation.^[19]^ The CKD-EPI equation without the race factor was utilized for estimating GFR in this study given that recent studies^[20–22]^ have suggested that the inclusion of race may generate less precise estimates in SSA. Participants’ kidney function was staged using the Kidney Disease outcome quality initiative classification.^[23]^ The prevalence (and 95% confidence interval) of CKD in the overall population and subgroups among the HIV patients was computed. To increase the power to generate stable estimates over time, the entire time of observation was divided into 5 time periods of 3 years each. The Cochran-Armitage trend test was used to assess for the presence of linear trends in CKD prevalence while the Cuzick trend test was used to determine linear trend in median CD4 count over the study period. Multivariable logistic regression models (using a threshold significance of *P*-value <.25 in univariable analyses and known CKD risk factors) were used to identify independent predictors of CKD in the study population. Three sets of sensitivity analyses were performed using eGFR estimates with the race factor for the CKD-EPI equation; eGFR estimates using the 4-variable Modification of Diet in Renal Disease equation with and without the race factor; and eGFR estimates using the CKD-EPI equation without the race factor for the population with at least 1 serum creatinine record.

## 3 Results

### 3.1 Demographic and clinical features

A total of 6676 patients had at least 1 GFR estimate. Of these, 1317 had 2 GFR estimates performed 3 or more months apart.

Table 1 summarizes the demographic and clinical characteristics of included patients. From the sample of 1317 patients, 62.2% were female. The mean age at enrolment was 35.4 ± 9.5 years; median CD4 count was 194 (interquartile range 95–343) cells/μL; and 51.1% had CD4 count lower than 200 cells/μL. Hypertension was present in 43.6% (95% CI: 41.5%–49.9%) and DM in 8.8% (95% CI: 5.6%–12.9%). Obesity (BMI > 30 kg/m^2^) was seen in 8.3% (95% CI: 6.7%–10.1%) of the study population, and overweight (BMI 25.0–29.9 kg/m^2^) in 21.9%. Dyslipidemia was present in 28.8% (95% CI 26.3%–38.8%), hepatitis B in 5.7%, and hepatitis C infection in 2.2%.

**Table 1:**
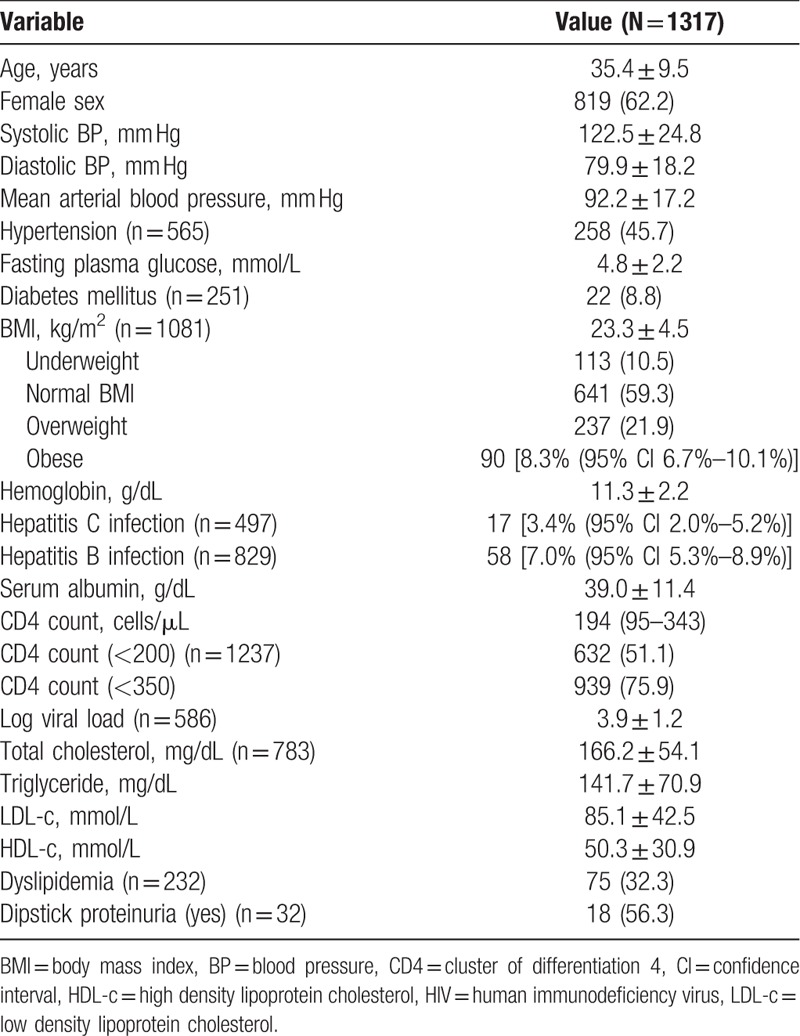
Demographic and clinical characteristics of HIV positive patients with at least 2 measurements of glomerular filtration rate.

### 3.2 Prevalence of CKD

**Figure 2:**
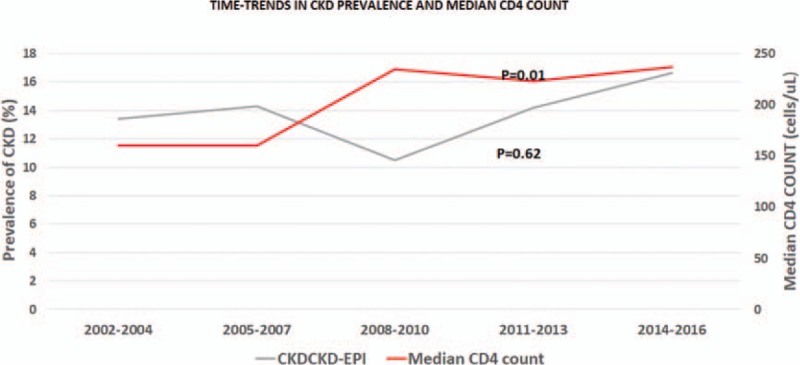
Relationship between CKD prevalence and median CD4 count (2002–2016). CD4 = cluster of differentiation 4, CKD = chronic kidney disease.

Of the 1317 patients who had at least 2 creatinine measures (at initiation of care and 3 months or more apart), the prevalence of CKD using the CKD-EPI equation without race was 13.4% (95% CI: 11.6%–15.4%). There was no significant change in CKD prevalence between 2002 and 2016, *P* = .62 (Fig. 2) while the median CD4 count at presentation progressively increased over the same period (*P* = .01).

The prevalence of CKD (stages 3, 4, and 5) was 8.8%, 2.2%, and 2.4%, respectively. There was no significant gender difference in the CKD prevalence (male vs female prevalence of 7.8% vs 9.4% for stage 3; 2.0% vs 2.3% for stage 4; and 2.6% vs 2.3% for stage 5, *P* = .75). Those with advanced CKD (stages 4 and 5) constituted 4.6% of the total population.

### 3.3 Sensitivity analyses of GFR equation estimates

Using the CKD-EPI equation with the race factor, the prevalence of CKD was 8.9% (95% CI: 7.5%–10.6%). The proportion of patients with stages 3, 4, and 5 CKD was 4.9%, 1.9%, and 2.1%, respectively. The prevalence of advanced CKD (stages 4 and 5) was 4.0% for CKD-EPI equation (with race factor).

The modification of diet in renal disease equation without the race factor yielded a CKD prevalence of 15.9% (95% CI: 13.9%–17.9%) with stages 3, 4, and 5 constituting 11.1%, 2.4%, and 2.4%, respectively. With the race factor, stages 3, 4, and 5 accounted for 5.5%, 1.9%, and 1.97%, respectively, making up 9.3% of the study population.

Using the single eGFR at initiation of care, a CKD prevalence of 26.6% (95% CI 25.6%–27.7%) with stages 3, 4, and 5 being 20.3%, 3.1%, and 3.2%, respectively, using the CKD-EPI equation without the race factor. Using the CKD-EPI equation with the race factor, stages 3, 4, and 5 had prevalence of 12.9%, 2.5%, and 2.9% respectively summing up to a CKD prevalence of 18.3%.

### 3.4 Factors associated with CKD

On multivariable analysis, increasing age was associated with increased risk of developing CKD – odds ratio (OR) 1.07 (95% CI: 1.05–1.10; *P* < .001) (Table 2). HIV patients with CD4 count less than 200 cells/μL were also at increased risk of developing CKD (Table 2). The presence of DM, hepatitis B and C coinfection, and hypertension did not increase CKD risk at the multivariable level. Comparison of sociodemographic and clinical characteristics of those with 1 and 2 GFR estimates (Supplementary Table 1) showed statistical (but not clinically relevant) difference in age; higher proportion of hypertension and DM among those with 2 GFR estimates and similar BMI, hemoglobin, and hepatitis B coinfection prevalence.

**Table 2:**
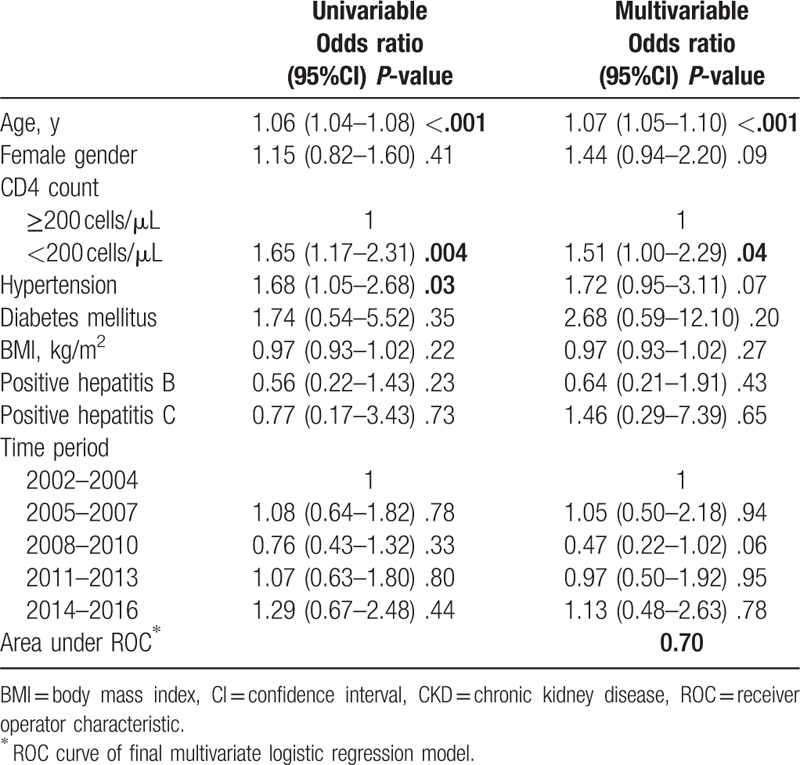
Multivariate analysis of the predictors of CKD in HIV positive patients in the Niger-Delta region of Southern Nigeria.

## 4 Discussion

This study is one of the few attempts, in a large HIV population in SSA, to report the prevalence of CKD at initiation of care. The main findings and therefore importance of this study are: showing a high prevalence of CKD in a population of HIV positive patients; identification of increasing age and low CD4 count as independent CKD risk factors in our HIV population.

The prevalence of CKD in our population of HIV positive patients is high. Several studies among HIV patients in Southern Nigeria^[11,12,24,25]^ with similar sociodemographic characteristics have also shown high prevalence of HIV individuals with eGFR < 60 mL/min/1.73 m^2^ in ART-naïve patients. Some have reported prevalence as high as 47.6%^[12]^ to 53%^[24]^ when proteinuria was also considered, even though persistent proteinuria was not demonstrated in those studies and single eGFR measures were used which cannot discriminate between CKD and AKI patients. Other studies have equally reported a high prevalence of CKD among ART-naïve individuals in the West African sub-region such as Ghana (38.8%)^[26]^ and Cameroon (54%).^[27]^ In other African regions, the prevalence of CKD in HIV positive patients is variable with South Africa recording 2%^[28,29]^ and Kenya 12%.^[30]^ Overall, it has been shown that blacks (in SSA or other parts of the world) have higher risk of CKD when they are HIV-infected.^[31]^

A number of factors complicate the HIV-CKD interplay in Africa. First is the suboptimal implementation of available guidelines^[32]^ for the screening, diagnosis, and management of CKD in HIV populations, especially in Africa. In our study population, less than 2% had dipstick proteinuria performed at initiation of care and none had proteinuria quantified either by spot or 24-hour urine collection. A recent report of the Global Kidney Health Atlas exploring global access of patients to health technologies and medications corroborates our finding by showing that African countries, of all world regions, had the lowest capacity for identification, monitoring, and management of CKD.^[33]^ Secondly, there is a need to develop CKD risk assessment tools that aid the identification of high risk HIV patients for screening and initiation of appropriate therapies. Of the few available CKD risk assessment tools, one was derived from a predominantly Caucasian HIV population which differs significantly according to demographics from the HIV populations in SSA.^[34]^ This tool has also not been validated in SSA HIV populations. Thirdly, GFR estimation formulae have not been properly validated in the HIV population. The few studies that have attempted doing this^[35,36]^ either had very small sample sizes or were performed in a restricted population, making the results not generalizable. This leaves room for inconsistencies and great variations in the diagnosis of CKD among HIV-infected individuals. Recently, some workers in West Africa^[20]^ have proposed the use of the full age spectrum serum creatinine-based equations constructed and validated by Pottel et al^[37]^ in Caucasian populations. The best creatinine-based eGFR equation is yet to be determined for the African HIV population and considerable variations exist in the proportion of CKD among the HIV population depending on the equation used.

Late presentation of patients to the HIV clinics may also have contributed to the high prevalence of CKD in our study population. Many patients do not routinely go to hospital for treatment in Nigeria due to the cost (out-of-pocket payment) associated with health care. In this study, late presentation is supported by the relatively low CD4 counts of these patients at time of their first visit. A low CD4 count has also been documented as a risk factor for CKD in other HIV populations^[38,39]^ probably mirroring more severe HIV disease, longer duration of exposure to HIV or presence of opportunistic infections all of which may predispose to both AKI and CKD. Increasing age and low CD4 counts were independent predictors of occurrence of CKD. Increasing age is a known risk factor for CKD in the general population and in the HIV population.^[39]^ Recurrent diarrheal disease in HIV patients not having adequate care may also lead to repeated episodes of undiagnosed acute kidney injury which may ultimately lead to CKD. Other cultural factors like use of herbal remedies with unproven efficacy for HIV cure but with known nephrotoxic potential may also contribute to the increased CKD occurrence in HIV patients in West Africa. These factors were however not assessed by this study.

Although our study did not assess genetic factors, the increased prevalence of CKD in people of West African descent with HIV compared to other parts of the world may suggest a genetic predisposition.^[40]^ Studies have reported that the presence of G1 and G2 high risk alleles of the apolipoprotein (APOL)1 gene is associated with HIVAN in South Africa.^[41,42]^ High risk alleles of APOL1 have been documented as risk factors among nondiabetic CKD patients in South East Nigeria,^[43]^ including a small group of HIVAN patients. Individuals with both APOL1 risk alleles have an estimated 4% lifetime risk for developing focal segmental glomerulosclerosis (FSGS), and untreated HIV-infected individuals have a 50% risk for developing HIVAN.^[44]^ On the other hand, HIV positive patients without the risk variants have negligible risk of developing HIVAN as documented in the Ethiopian population.^[45]^ A gene–environment interaction mediated by interferons and other cytokines (elaborated by the HIV infection) has been suggested as one of the factors leading to high CKD risk.^[46]^ There is also the possibility of gene–gene interactions increasing the risk of CKD progression in HIV patients of West African origin suggesting the need for investigation of other genetic risk factors for CKD progression among HIV patients.

The large proportion of patients for whom urine protein was not assessed nor serum creatinine repeated suggests significant gaps in both the evaluation and care offered to HIV patients. It may be necessary to set up prospective studies in a more controlled setting to evaluate the real estimates of chronic kidney dysfunction.

Most of the patients with CKD in our study population were in stage 3 disease where there is still a window of opportunity to slow down or stop progression to end-stage kidney disease by early initiation of ART and other renal-specific interventions such as the use of ACE-inhibitors (or angiotensin receptor blockers) to reduce proteinuria and control of blood pressure. Early detection and retarding of CKD progression through initiation of appropriate therapies may be more cost effective at retarding mortality given that access to treatment of advanced CKD is limited and expensive.^[47]^

### 4.1 Limitations

A limitation of this study includes the retrospective design as medical records of some patients who were registered at the HIV clinic were unavailable for assessment and inclusion into this study. However, given the large sample size of our study, we believe patients included are an adequate representation of the enrolled patients. Another study limitation relates to the unavailability of albuminuria. Our definition of CKD therefore depended only on serum creatinine and eGFR which could underestimate CKD prevalence. Studies reporting CKD prevalence using eGFR and albuminuria^[5,6]^ generally tend to have higher prevalence rates than if CKD was diagnosed with eGFR only. This study is however strengthened by the large sample size and by utilizing various GFR estimation equations for assessing CKD. The large sample size increases confidence in the reported prevalence rates. The inclusion of patients across many years of activities has provided the opportunity of assessing the time trends.

## 5 Conclusion

This study reports a high prevalence of CKD in HIV positive patients in Nigeria. Earlier presentation of patients and earlier initiation of treatment could contribute to further reduction in the prevalence of disease. Strategies toward earlier identification of HIV positive patients at risk of CKD are therefore needed to reduce CKD burden among HIV patients in SSA. It is also important to determine the best serum creatinine-based GFR estimating equation for HIV patients in SSA.

## Acknowledgments

The authors thank the staff of the UUTH HIV clinic, and fhi360 for providing access to the clinic database and the Batch VIII medical students for helping with data collection.

## Author contributions

**Conceptualization:** Udeme Ekpenyong Ekrikpo, Andre Pascal Kengne, Ikechi Okpechi.

**Data curation:** Udeme Ekpenyong Ekrikpo, Effiong Ekong Akpan, Emmanuel Effa, John Ekott.

**Formal analysis:** Udeme Ekpenyong Ekrikpo, Andre Pascal Kengne, Ikechi Okpechi.

**Methodology:** Udeme Ekpenyong Ekrikpo, Andre Pascal Kengne, Effiong Ekong Akpan, Emmanuel Effa, Aminu Bello, John Ekott, Cindy George, Babatunde Salako, Ikechi Okpechi.

**Project administration:** Udeme Ekpenyong Ekrikpo, Ikechi Okpechi.

**Resources:** Udeme Ekpenyong Ekrikpo, Andre Pascal Kengne, Cindy George, Babatunde Salako.

**Supervision:** Aminu Bello, John Ekott, Ikechi Okpechi.

**Validation:** Andre Pascal Kengne, Ikechi Okpechi.

**Writing – original draft:** Udeme Ekpenyong Ekrikpo.

**Writing – review & editing:** Udeme Ekpenyong Ekrikpo, Andre Pascal Kengne, Effiong Ekong Akpan, Emmanuel Effa, Aminu Bello, John Ekott, Cindy George, Babatunde Salako, Ikechi Okpechi.

## Supplementary Material

Supplemental Digital Content

## References

[1] UNAIDS. Global AIDS Update 2016. Geneva: UNAIDS; 2016.

[2] National Agency for the Control of AIDS (NACA). Global AIDS response; country progress report Nigeria 2014. Abuja: NACA; 2014.

[3] UNAIDS. The GAP Report. Geneva: UNAIDS; 2014.

[4] Samji H Cescon A Hogg RS Closing the gap: increases in life expectancy among treated HIV-positive individuals in the United States and Canada. PloS One 2013;8:e81355. 24367482 10.1371/journal.pone.0081355 PMC3867319

[5] Gupta SK Mamlin BW Johnson CS Prevalence of proteinuria and the development of chronic kidney disease in HIV-infected patients. Clin Nephrol 2004;61:1–6. 14964451 10.5414/cnp61001

[6] Wyatt CM Winston JA Malvestutto CD Chronic kidney disease in HIV infection: an urban epidemic. AIDS (Lond, Engl) 2007;21:2101–3. 10.1097/QAD.0b013e3282ef1bb4 17885301

[7] Okpechi IG Rayner BL Swanepoel CR Nephrotic syndrome in adult black South Africans: HIV-associated nephropathy as the main culprit. J Natl Med Assoc 2010;102:1193–7. 21287900 10.1016/s0027-9684(15)30774-4

[8] Akpan EE Ekrikpo UE Udo AIA Changing aetiogies of end stage kidney disease in a resource poor enivornment: what is the way forward? World J Biomed Res 2014;1:1–5.

[9] Davids M Marais N Jacobs J South African Renal Registry Annual Report 2012 2012.

[10] Razzak Chaudhary S Workeneh BT Montez-Rath ME Trends in the outcomes of end-stage renal disease secondary to human immunodeficiency virus-associated nephropathy. Nephrol Dial Transplant 2015;30:1734–40. 26175146 10.1093/ndt/gfv207 PMC4829059

[11] Anyabolu EN Chukwuonye II Arodiwe E Prevalence and predictors of chronic kidney disease in newly diagnosed human immunodeficiency virus patients in Owerri, Nigeria. Indian J Nephrol 2016;26:10–5. 26937072 10.4103/0971-4065.156115 PMC4753735

[12] Ayokunle DS Olusegun OT Ademola A Prevalence of chronic kidney disease in newly diagnosed patients with Human immunodeficiency virus in Ilorin, Nigeria. J Bras Nefrol 2015;37:177–84. 26154637 10.5935/0101-2800.20150029

[13] Umeizudike T Mabayoje M Okany C Prevalence of chronic kidney disease in HIV positive patients in Lagos, south-west Nigeria. Nephrol Rev 2012;4:22–6.

[14] Agaba EI Agaba PA Sirisena ND Renal disease in the acquired immunodeficiency syndrome in north central Nigeria. Niger J Med 2003;12:120–5. 14737980

[15] Chobanian AV Bakris GL Black HR The Seventh Report of the Joint National Committee on Prevention, Detection, Evaluation, and Treatment of High Blood Pressure: the JNC 7 report. JAMA 2003;289:2560–72. 12748199 10.1001/jama.289.19.2560

[16] ADA. Diagnosis and classification of diabetes mellitus. Diabetes Care 2010;33 (Supplement 1):S62–9. 20042775 10.2337/dc10-S062 PMC2797383

[17] World Health Organization. Obesity: Preventing and Managing the Global Epidemic. Report of a WHO consultation. Geneva: WHO; 2000. 11234459

[18] Williams L Third report of the National Cholesterol Education Program (NCEP) expert panel on detection, evaluation, and treatment of high blood cholesterol in adults (Adult Treatment Panel III) final report. Circulation 2002;106:3143–13143. 12485966

[19] Levey AS Stevens LA Schmid CH A new equation to estimate glomerular filtration rate. Ann Intern Med 2009;150:604–12. 19414839 10.7326/0003-4819-150-9-200905050-00006 PMC2763564

[20] Yayo E Ayé M Yao C Measured (and estimated) glomerular filtration rate: reference values in West Africa. Nephrol Dial Transplant 2017;doi: 10.1093/ndt/gfx244. 10.1093/ndt/gfx244 28992086

[21] Anker N Scherzer R Peralta C Racial disparities in creatinine-based kidney function estimates among HIV-infected adults. Ethn Dis 2016;26:213–20. 27103772 10.18865/ed.26.2.213 PMC4836902

[22] van Deventer HE George JA Paiker JE Estimating glomerular filtration rate in black South Africans by use of the modification of diet in renal disease and Cockcroft-Gault equations. Clin Chem 2008;54:1197–202. 18487286 10.1373/clinchem.2007.099085

[23] Levey AS Coresh J Bolton K K/DOQI clinical practice guidelines for chronic kidney disease. Am J Kidney Dis 2002;39 (2 Suppl 1): 11904577

[24] Okafor UH Unuigbe EI Chukwuonye E Prevalence and clinical and laboratory characteristics of kidney disease in anti-retroviral-naive human immunodeficiency virus-infected patients in South-South Nigeria. Saudi J Kidney Dis Transpl 2016;27:129–34. 26787579 10.4103/1319-2442.174155

[25] Emem CP Arogundade F Sanusi A Renal disease in HIV-seropositive patients in Nigeria: an assessment of prevalence, clinical features and risk factors. Nephrol Dial Transplant 2008;23:741–6. 18065807 10.1093/ndt/gfm836

[26] Sarfo FS Keegan R Appiah L High prevalence of renal dysfunction and association with risk of death amongst HIV-infected Ghanaians. J Infect 2013;67:43–50. 23542785 10.1016/j.jinf.2013.03.008

[27] FolefackKaze F Kengne AP Pefura Yone EW Renal function, urinalysis abnormalities and correlates among HIV-infected Cameroonians naive to antiretroviral therapy. Saudi J Kidney Dis Transpl 2013;24:1291–7. 24231506 10.4103/1319-2442.121280

[28] Wensink GE Schoffelen AF Tempelman HA Albuminuria is associated with traditional cardiovascular risk factors and viral load in HIV-infected patients in rural South Africa. PLoS One 2015;10:e0136529. 26309226 10.1371/journal.pone.0136529 PMC4550462

[29] Kamkuemah M Kaplan R Bekker LG Renal impairment in HIV-infected patients initiating tenofovir-containing antiretroviral therapy regimens in a primary healthcare setting in South Africa. Trop Med Int Health 2015;20:518–26. 25442109 10.1111/tmi.12446

[30] Edwards JK Bygrave H Van den Bergh R HIV with non-communicable diseases in primary care in Kibera, Nairobi, Kenya: characteristics and outcomes 2010–2013. Trans R Soc Trop Med Hyg 2015;109:440–6. 25997923 10.1093/trstmh/trv038

[31] Lucas GM Lau B Atta MG Chronic kidney disease incidence, and progression to end-stage renal disease, in HIV-infected individuals: a tale of two races. J Infect Dis 2008;197:1548–57. 18422458 10.1086/587994 PMC2553209

[32] Lucas GM Ross MJ Stock PG Clinical practice guideline for the management of chronic kidney disease in patients infected with HIV: 2014 update by the HIV Medicine Association of the Infectious Diseases Society of America. Clin Infect Dis 2014;59:e96–38. 25234519 10.1093/cid/ciu617 PMC4271038

[33] Htay H Alrukhaimi M Ashuntantang GE Global access of patients with kidney disease to health technologies and medications: findings from the Global Kidney Health Atlas project. Kidney Int Suppl 2018;8:64–73. 10.1016/j.kisu.2017.10.010 PMC6336224 30675440

[34] Mocroft A Lundgren JD Ross M Development and validation of a risk score for chronic kidney disease in HIV infection using prospective cohort data from the D:A:D study. PLoS Med 2015;12:e1001809. 25826420 10.1371/journal.pmed.1001809 PMC4380415

[35] Barraclough K Er L Ng F A comparison of the predictive performance of different methods of kidney function estimation in a well-characterized HIV-infected population. Nephron Clin Pract 2009;111:c39–48. 19052469 10.1159/000178978

[36] Praditpornsilpa K Avihingsanon A Chaiwatanarat T Comparisons between validated estimated glomerular filtration rate (GFR) equations and isotopic GFR in HIV patients. AIDS (Lond, Engl) 2012;26:1781–8. 10.1097/QAD.0b013e328356480d PMC3782632 22713478

[37] Pottel H Hoste L Dubourg L An estimated glomerular filtration rate equation for the full age spectrum. Nephrol Dial Transpl 2016;31:798–806. 10.1093/ndt/gfv454 PMC4848755 26932693

[38] da Silva DR Gluz IC Kurz J Multiple facets of HIV-associated renal disease. Braz J Med Biol Res 2016;49:e5176. 27007656 10.1590/1414-431X20165176 PMC4819412

[39] Cheung CY Wong KM Lee MP Prevalence of chronic kidney disease in Chinese HIV-infected patients. Nephrol Dial Transpl 2007;22:3186–90. 10.1093/ndt/gfm350 17575315

[40] Mallipattu SK Salem F Wyatt CM The changing epidemiology of HIV-related chronic kidney disease in the era of antiretroviral therapy. Kidney Int 2014;86:259–65. 24573317 10.1038/ki.2014.44

[41] Kasembeli AN Duarte R Ramsay M APOL1 risk variants are strongly associated with HIV-associated nephropathy in Black South Africans. J Am Soc Nephrol 2015;26:2882–90. 25788523 10.1681/ASN.2014050469 PMC4625661

[42] Kasembeli AN Duarte R Ramsay M African origins and chronic kidney disease susceptibility in the human immunodeficiency virus era. World J Nephrol 2015;4:295–306. 25949944 10.5527/wjn.v4.i2.295 PMC4419140

[43] Ulasi II Tzur S Wasser WG High population frequencies of APOL1 risk variants are associated with increased prevalence of non-diabetic chronic kidney disease in the Igbo people from south-eastern Nigeria. Nephron Clin Pract 2013;123:123–8. 23860441 10.1159/000353223

[44] Kopp JB Nelson GW Sampath K APOL1 genetic variants in focal segmental glomerulosclerosis and HIV-associated nephropathy. J Am Soc Nephrol 2011;22:2129–37. 21997394 10.1681/ASN.2011040388 PMC3231787

[45] Behar DM Shlush LI Maor C Absence of HIV-associated nephropathy in Ethiopians. Am J Kidney Dis 2006;47:88–94. 16377389 10.1053/j.ajkd.2005.09.023

[46] Nichols B Jog P Lee JH Innate immunity pathways regulate the nephropathy gene apolipoprotein L1. Kidney Int 2015;87:332–42. 25100047 10.1038/ki.2014.270 PMC4312530

[47] Okpechi IG ESKD in sub-Saharan Africa: will governments now listen? Lancet Glob Health 2017;5:e373–4. 28229925 10.1016/S2214-109X(17)30070-0

