# Supplementary material for: Prevalence and correlates of chronic kidney disease (CKD) among ART-naive HIV patients in the Niger-Delta region of Nigeria
Source: Medicine (Baltimore). 2018 Apr 20;97(16):e0380. doi: 10.1097/MD.0000000000010380 (PMC5916672; doi:10.1097/MD.0000000000010380)
Supplement: Supplemental Digital Content [file medi-97-e0380-s001.docx]

**Supplementary table 1: Demographic and clinical characteristics of all HIV patients**

|  | **Total** | **One serum creatinine measure** | **Two serum creatinine measures** | **p** |
| --- | --- | --- | --- | --- |
| **Age (years)** | 34.7±9.8 | 34.5±9.9 | 35.4±9.5 | 0.004 |
| **Female Sex** | 4024 (60.3) | 3205 (59.8) | 819 (62.2) | 0.11 |
| **Systolic BP (mmHg)** | 117.4 ± 24.1 | 116.2±23.7 | 122.5±24.8 | <0.001 |
| **Diastolic BP (mmHg)** | 77.9 ± 17.9 | 77.4±17.7 | 79.9±18.2 | 0.003 |
| **MABP (mmHg)** | 89.1 ± 16.7 | 88.4±16.4 | 92.2±17.3 | <0.001 |
| **Hypertension (n=2909)** | 1018 [95%CI 35.0% (33.3–36.8%)] | 760 (32.8) | 258 (45.7) | <0.001 |
| **FPG (mmol/L)** | 4.6 ± 2.1 | 4.6±2.0 | 4.8±2.2 | 0.21 |
| **Diabetes Mellitus (n=1506)** | 83 [5.5% (4.4 – 6.8%)] | 61 (4.9) | 22 (8.8) | 0.01 |
| **BMI (kg/m^2^) (n= 3859)** | 23.1 ± 4.6 | 23.0±4.6 | 23.3±4.5 | 0.07 |
| **Underweight**  **Normal BMI**  **Overweight**  **Obese** | 451 (11.7)  2252 (58.4)  855 (22.2)  301 [7.8% (7.0 – 8.7%)] | 338 (12.2)  1611 (58.0)  618 (22.3)  211 (7.6) | 113 (10.5)  641 (59.3)  237 (21.9)  90 (8.3) | 0.43 |
| **Hb (g/dl) (n = 1031)** | 11.0 ± 2.4 | 10.9±2.5 | 11.3±2.2 | 0.06 |
| **HCV co-infection (n = 2489)** | 55 [2.2% (1.7–2.9%)] | 38 (1.9) | 17 (3.4) | 0.04 |
| **HBV co-infection (n = 3919)** | 225 [5.7% (5.0–6.5%)] | 167 (5.4) | 58 (7.0) | 0.08 |
| **Serum albumin (g/dl)** | 39.8 ± 13.6 | 39.9±14.1 | 39.0±11.4 | 0.38 |
| **CD4 count (cells/ul)** | 192 (88–349) | 191 (84-351) | 194 (95-343) | 0.38 |
| **CD4 count (<200) (n=6047)** | 3093 (51.1%) | 2461 (51.2) | 632 (51.1) | 0.96 |
| **CD4 count (<350)** | 4537 (75.1%) | 3598 (74.9) | 939 (75.9) | 0.45 |
| **Log Viral load (n=586)** | 3.7±1.2 | 3.6±1.2 | 3.9±1.2 | 0.02 |
| **Total Chol (mmol/L) (n=783)** | 4.1 ± 1.3 | 4.1±1.2 | 4.3±1.4 | 0.02 |
| **Triglyceride (mmol/L)** | 1.6 ± 0.8 | 1.6±0.9 | 1.6±0.8 | 0.81 |
| **LDL-c (mmol/L)** | 2.1 ± 0.9 | 2.0±0.9 | 2.2±1.1 | 0.25 |
| **HDL-c (mmol/L)** | 1.3 ± 0.8 | 1.3±0.8 | 1.3±0.8 | 0.97 |
| **Dyslipidemia (n=823)** | 237 [28.8% (25.7–32.0%)] | 162 (27.4) | 75 (32.3) | 0.16 |
| **Dipstick Proteinuria (n=123)** | 62 [50.4% (41.2–59.5%)] | 44 (48.4) | 18 (56.3) | 0.44 |

*BP=blood pressure; MABP= mean arterial blood pressure; FPG=fasting plasma glucose; BMI = Body mass index; Hb=Haemoglobin; HCV=hepatitis C virus; HBV=hepatitis B virus; Total Chol=total cholesterol; LDL-c = Low Density lipoprotein; HDL-c = High Density lipoprotein*
